# Virtual navigation bronchoscopy‐guided intraoperative indocyanine green localization in simultaneous surgery for multiple pulmonary nodules

**DOI:** 10.1111/1759-7714.14633

**Published:** 2022-09-04

**Authors:** Qingjie Yang, Kaibao Han, Shenghua Lv, Qingtian Li, Xiaoyan Sun, Xinhai Feng, Mingqiang Kang

**Affiliations:** ^1^ Department of Thoracic Surgery Fujian Medical University Union Hospital Fuzhou P. R. China; ^2^ Department of Thoracic Surgery Xiamen Humanity Hospital of Fujian Medical University Xiamen P. R. China; ^3^ Key Laboratory of Cardio‐Thoracic Surgery (Fujian Medical University) Fujian Province University Fuzhou China; ^4^ Key Laboratory of Ministry of Education for Gastrointestinal Cancer Fujian Medical University Fuzhou China; ^5^ Fujian Key Laboratory of Tumor Microbiology Fujian Medical University Fuzhou China

**Keywords:** intraoperative localization, multiple pulmonary nodules, simultaneous surgery, virtual navigation bronchoscopy, wedge resection

## Abstract

**Background:**

Accurate localization of pulmonary nodules is the main difficulty experienced in wedge resection. Commonly used localization methods have their own advantages and disadvantages. However, clinical work has demonstrated that intraoperative indocyanine green localization under electromagnetic navigation bronchoscopy/virtual navigation bronchoscopy (VNB) is more advantageous than conventional methods for patients with multiple pulmonary nodules undergoing simultaneous surgery, especially for those undergoing bilateral lung surgery.

**Methods:**

Data of patients undergoing simultaneous surgery for multiple pulmonary nodules with preoperative methylene blue localization by computed tomography (CT)‐guided percutaneous lung puncture (methylene blue group) or intraoperative indocyanine green localization under VNB (virtual navigation group) were retrospectively analyzed. Patient characteristics, pulmonary nodule features, localization time, preoperative location time, location success rate, operation time, complication incidence, visceral pleural staining rate after localization, and pulmonary nodule primary resection success rate were compared between the two groups.

**Results:**

The methylene blue and virtual navigation groups comprised 39 and 20 patients with 119 and 67 pulmonary nodules resected, respectively. Sex, age, number of pulmonary nodules resected simultaneously, unilateral/bilateral lung surgery, pulmonary nodule size, distance between pulmonary nodules and the visceral pleura, pulmonary nodule consolidation‐to‐tumor ratio, location of pulmonary nodules in the pulmonary lobe, postoperative pathology, visceral pleura staining rate, primary pulmonary nodule resection success rate, and surgical duration did not differ significantly between the groups (*p* > 0.05). The localization time of the virtual navigation group was significantly shorter than that of the methylene blue group (*p* < 0.05), regardless of unilateral or bilateral multiple nodules. In the methylene blue group, 25.64% (10/39) of patients presented complications, all of which were pneumothorax, whereas no complications were found in the virtual navigation group.

**Conclusions:**

For patients with multiple pulmonary nodules undergoing simultaneous surgery, indocyanine green injection under VNB can achieve a similar effect on pulmonary nodule localization as classical methylene blue injection under CT‐guided percutaneous lung puncture, with shorter localization time and fewer complications.

## Graphical abstract

For multiple pulmonary nodules undergoing simultaneous surgery, indocyanine green injection under virtual navigation bronchoscopy can achieve a similar effect as classical methylene blue injection under computed tomography‐guided percutaneous lung puncture, with shorter localization time and fewer complications.

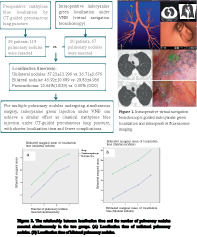

**Abbreviations**
ENB: electromagnetic navigation bronchoscopy
VNB: virtual navigation bronchoscopy
AAH: atypical adenomatous hyperplasia
AIS: adenocarcinoma in situ
MIA: minimally invasive adenocarcinoma
CTR: consolidation‐to‐tumor ratio
CT: computed tomography
DICOM: digital imaging and communications in medicine
3D: three dimensional

## INTRODUCTION

The popularization of chest computed tomography (CT) in routine physical examination has enabled many patients with early lung cancer manifesting as pulmonary nodules to be diagnosed. Pulmonary wedge resection is one of main surgical method for pulmonary nodules. However, the accurate localization of pulmonary nodules is the main difficulty experienced in wedge resection. At present, the commonly used pulmonary nodule localization methods include preoperative CT‐guided percutaneous lung puncture,^1^, ^2^ spring coil localization,^3^ methylene blue localization,^4^ X‐ray localization under thoracoscopy,^5^, ^6^ intraoperative ultrasonic localization,^7^, ^8^ intraoperative localization using infrared imaging,^9^, ^10^ biological glue injection,^11^, ^12^, ^13^, ^14^, ^15^ and intraoperative fluorescence localization under electromagnetic navigation bronchoscopy electromagnetic navigational bronchoscopy (ENB)/virtual navigation bronchoscope (VNB),^16^, ^17^, ^18^, ^19^ and others.^20^ Each localization method has its own advantages and disadvantages, but clinical work has demonstrated that intraoperative indocyanine green localization under ENB/VNB has more advantages for patients with multiple pulmonary nodules undergoing simultaneous surgery, especially for those undergoing bilateral lung surgery. Therefore, this study retrospectively analyzed the data of patients undergoing simultaneous surgery for multiple pulmonary nodules in our hospital between 2018 and 2022. In addition, the advantages and disadvantages of preoperative methylene blue localization under CT‐guided percutaneous lung puncture and intraoperative indocyanine green localization under VNB were compared.

## METHODS

This study was approved by the Medical Ethics Committee of Xiamen Hongai Hospital affiliated with Fujian Medical University (NO. 2022‐013). The study was conducted in accordance with the provisions of the Declaration of Helsinki. Considering its retrospective design, the requirement of informed consent of each patient was waived by the ethics committee.

### Cases collected

**FIGURE 1:**
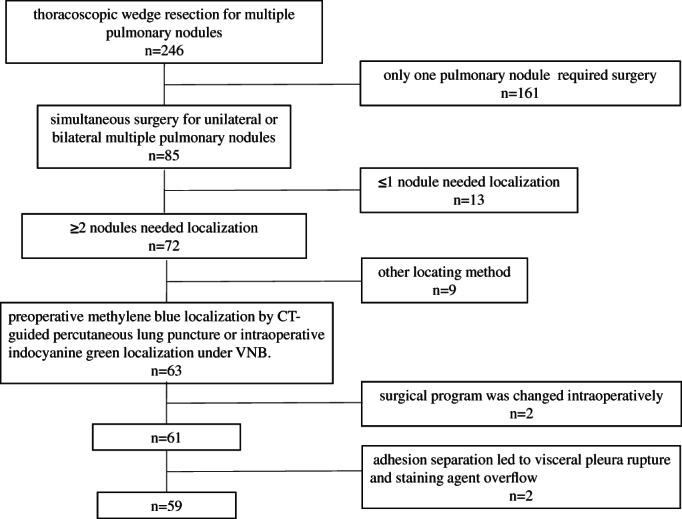
Cases collected

The data of patients undergoing thoracoscopic wedge resection for multiple pulmonary nodules in our hospital between January 1, 2019, and March 31, 2022, were collected. The inclusion criteria were as follows: (1) patients with multiple pulmonary nodules and more than two peripheral nodules clinically diagnosed as early lung cancer and requiring surgery; (2) patients undergoing simultaneous surgery for unilateral or bilateral multiple pulmonary nodules; (3) patients with more than two nodules needing wedge resection and localization; and (4) patients in whom all pulmonary nodules were to be resected and requiring preoperative methylene blue localization by CT‐guided percutaneous lung puncture or intraoperative indocyanine green localization under VNB. The exclusion criteria were as follows: (1) cases where the surgical program was changed intraoperatively after localization and wedge resection was not performed, instead of segmental/subsegmental lung resection or lobectomy; and (2) patients with a large‐area adhesion between the lung and the chest wall where the adhesion separation led to visceral pleura rupture and staining agent overflow, thereby affecting localization (Figure 1).

Based on the pulmonary nodule localization method, patients were divided into the methylene blue group (injection of methylene blue under CT‐guided percutaneous lung puncture) and the virtual navigation group (indocyanine green localization under VNB). The data of the enrolled patients are presented in Table 1.

**TABLE 1:** Baseline of the two groups

Observation indicators		Virtual navigation group (*x*¯ ± *s*)	Methylene blue group (*x*¯ ± *s*)	*t*/*χ* ^2^/*F*	*p*
Patients		20	39	–	–
Sex	Male(n)	10.0% (7/20)	33.33% (13/39)	0.674	0.412
	Female(n)	90.0% (18/20)	53.85% (21/39)		
				
Age (years)		44.95 ± 10.894	47.59 ± 15.068	−0.695	0.490
Number of pulmonary nodules resected simultaneously (*n*)		3.35 ± 1.182	3.05 ± 0.944	1.055	0.296
Unilateral/bilateral lung surgery (*n*)	Unilateral	70.0% (14/20)	69.23% (27/39)	0.004	0.952
	Bilateral	30.0% (6/20)	30.77% (12/39)		
Location of pulmonary nodules (*n*)	Right upper lung	20.90% (14/67)	21.85% (26/119)	2.595	0.628
	Right middle lobe	7.46% (5/67)	10.08% (12/119)		
	Right lower lobe	35.82% (24/67)	30.25% (36/119)		
	Left upper lung	17.91% (12/67)	25.21% (30/119)		
	Left lower lobe	17.91% (12/67)	12.61% (15/119)		
Pulmonary nodule size (mm)		9.299 ± 1.923	9.336 ± 2.100	−0.121	0.904
Distance between pulmonary nodules and the visceral pleura (mm)		13.970 ± 5.734	14.227 ± 6.364	−0.274	0.785
CTR (%)		0.050 ± 0.136	0.074 ± 0.163	−0.987	0.325

### Preoperative 3D reconstruction of lung tissue

**FIGURE 2:**
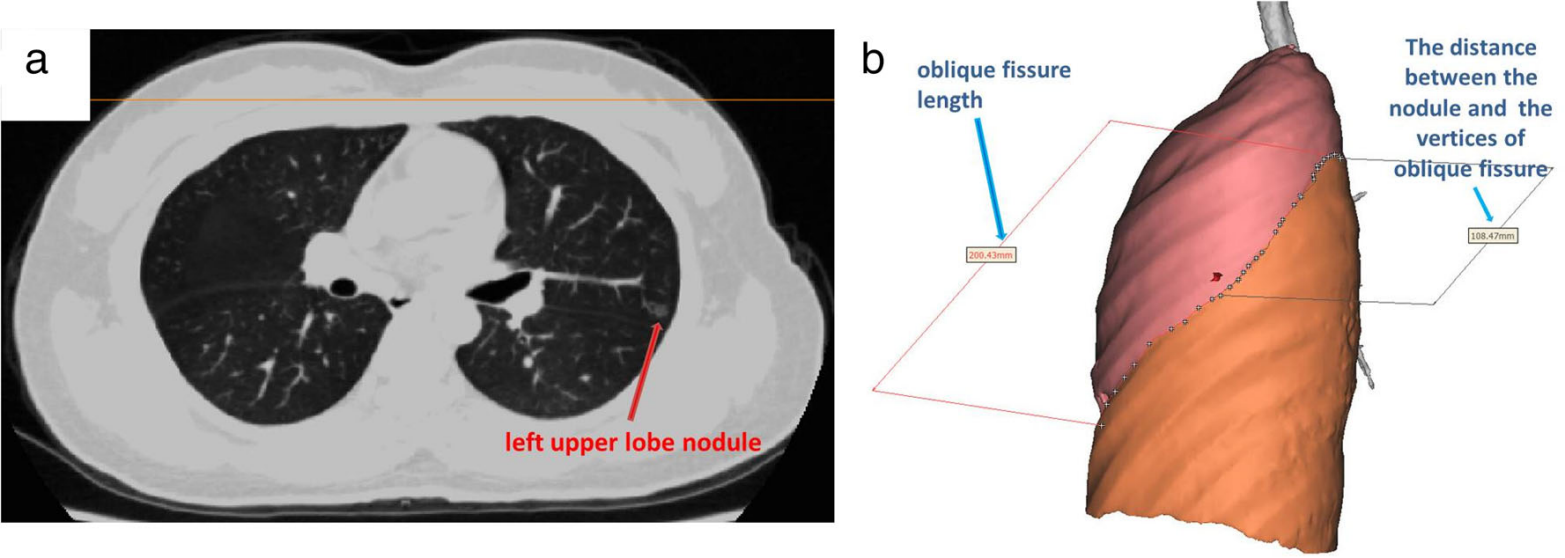
(a) Left upper pulmonary nodules, approximately 1 cm in diameter. (b) The distance between the projection point on the pulmonary surface and the edge of the oblique fissure was measured. The pulmonary nodules were located near the midpoint of the oblique fissure

Before surgery, the Digital Imaging and Communications in Medicine (DICOM) data of pulmonary thin‐slice CT were used for three‐dimensional (3D) reconstruction of the lung. These could be used for 3D lung surface projection to localize pulmonary nodules when methylene blue localization or virtual navigation localization failed. The lung on the affected side was 3D reconstructed using computer software (Xiamen Qiangben Technology Co., Ltd) to display its overall shape. The pulmonary lobes were segmented to show interlobar fissures. Subsequently, a 3D reconstruction of the pulmonary nodules was performed, showing their location in the lung. The projection of the pulmonary nodules on the lung surface was marked. The distances between the projection point and pulmonary nodules, as well as the visible anatomical features on the lung surface, such as the lung apex, horizontal fissure, and oblique fissure, were measured. Finally, the measured distance data were converted to ratios as much as possible, such as converting “at 5 cm from the inside to the outside along the horizontal fissure” to “at the intermediolateral 1/3 intersection of the horizontal fissure” for the intraoperative localization, as shown in Figure 2.

### Preoperative CT‐guided percutaneous lung piercing methylene blue localization

On the day of surgery, localization was performed in the CT room in the morning. Based on the approximate position of pulmonary nodules, patients adjusted their postures with the positioning paper (grid paper with lead lines) attached to the approximate position of the body surface projection of the pulmonary nodules. First, thin‐slice CT scanning was performed. Then, the optimal puncture point for localization was selected at 0.5 cm below the visceral pleura and 1 cm adjacent to the pulmonary nodule. After the puncture point, puncture depth, puncture angle, and chest wall thickness on the percutaneous puncture path were determined, and disinfection and towel paving were performed. Next, 5 ml of 2% lidocaine was extracted using a 5‐ml syringe equipped with an attached No. 5 long needle (0.5 mm × 60 mm). In addition, 0.05 ml of methylene blue (2 ml: 20 mg/piece; Jiangsu Qichuan Pharmaceutical Co., Ltd) and 0.95 ml of air were extracted using a 1‐ml syringe, and methylene blue was pushed to the front end of the syringe. According to the planned puncture angle, lidocaine was injected using the needle at the puncture point. Before lidocaine injection, the syringe was pulled back to remove any blood or air bubbles. After the puncture needle was estimated to penetrate the chest wall, lidocaine was injected and the needle was directly lowered to the predetermined depth. Subsequently, the chest was scanned using thin‐slice CT within 1 cm above and below the puncture level. After confirming that the puncture needle tip reached the predetermined position, the needle was connected to a 1‐ml syringe containing methylene blue. After all the methylene blue and air in the syringe were injected, the needle was withdrawn. Then, another thin‐slice CT scanning was performed on the chest within 1 cm above and below the puncture point level to clarify the relative position of methylene blue injected into the lung tissue and pulmonary nodules. Similarly, other nodules in the ipsilateral or contralateral lung were localized successively. After localization, if CT findings revealed remarkable hemopneumothorax (pneumothorax with lung compression >30% or estimated pleural hemorrhage >200 ml), closed drainage was performed at the planned surgical incision site (between the fourth and fifth intercostal space of the midaxillary line).

### Intraoperative VNB‐guided indocyanine green localization

**FIGURE 3:**
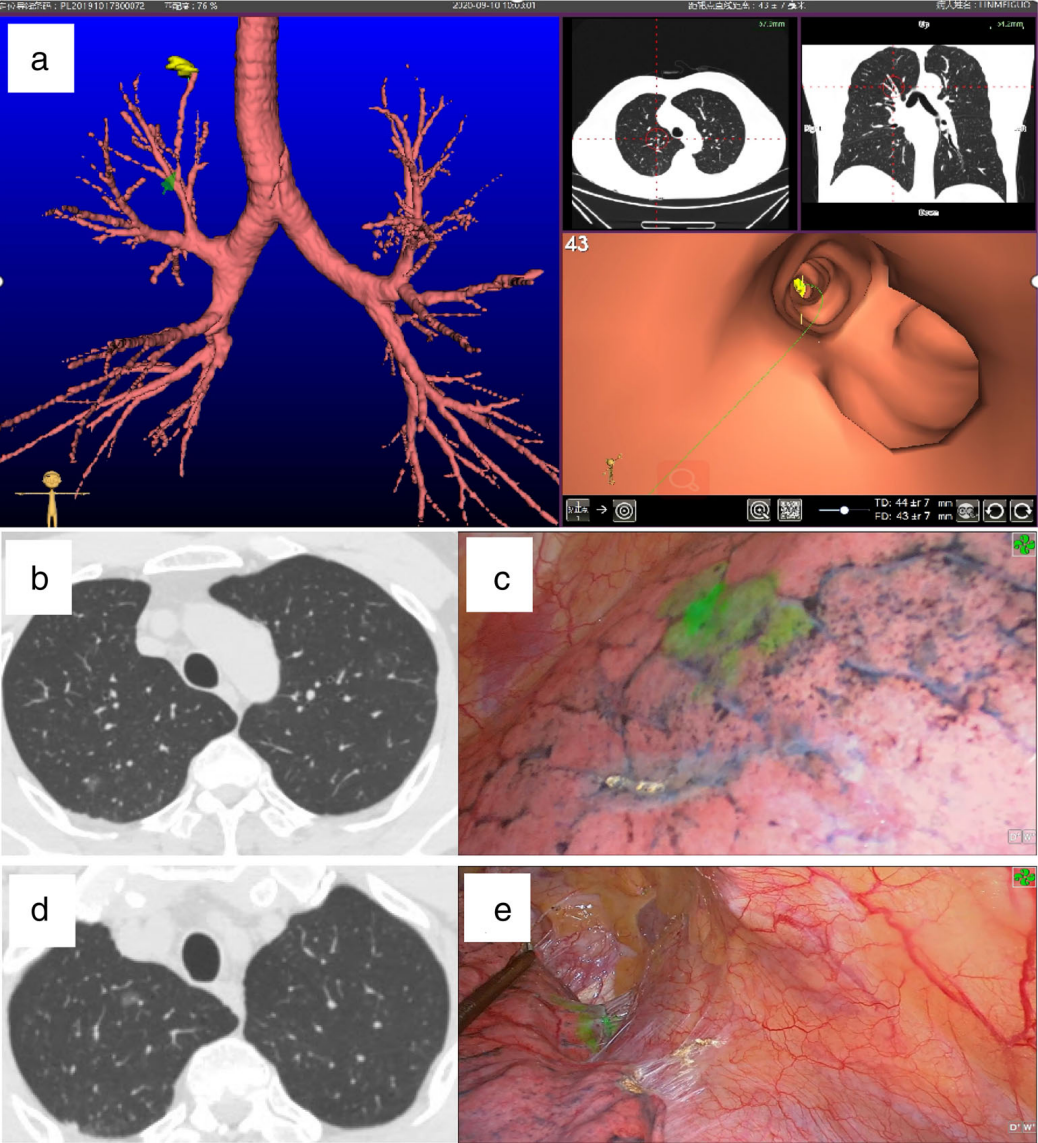
Intraoperative virtual navigation bronchoscopic‐guided indocyanine green localization and intraoperative fluorescence imaging. (a) Image of VNB. (b, c) CT and fluorescence images of a nodule in the posterior segment of the right upper lung. (d, e) CT and fluorescence images of a nodule in the apical segment of the right upper lung

The DICOM data of the preoperative chest thin‐slice CT (slice thickness <1 mm, slice spacing <1 mm) were introduced into the virtual navigation system (Lungpro augmented reality optical whole‐lung diagnosis and treatment navigation system, Kunbo Biological Technology Co., Ltd). The 3D reconstruction software of the system was used to extract bronchovascular bundles and reconstruct 3D maps of the lung and pulmonary nodules. The target pulmonary nodules were determined manually. The bronchoscopic route was generated by the software. After entering the operating room, patients were administered general anesthesia and single‐lumen No. 7.5 endotracheal intubation. While in the supine position, a 3.1 mm bronchoscope (flexible video bronchoscope BR‐1231, Zhuhai Seesheen Medical Technology Co., Ltd) was inserted through the endotracheal intubation. The bronchoscope image was transferred to the virtual navigation system, and the system automatically matched the real bronchoscope image and the 3D reconstructed image in real time, namely mixed virtual reality, and displayed the bronchoscope route in the field of view at the same time. The bronchoscope reached the target bronchus (usually the grade 4 bronchus) under the guidance of the virtual navigation system and entered the sheath tube through the biopsy channel for the bronchoscopy. The sheath tube was then directed to the grade 5 or 6 bronchus through the path navigated by the virtual software. Next, 0.3 ml of 0.6 mg/mL indocyanine green solution (25 mg/piece; Dandong Yichuang Pharmaceutical Co., Ltd) and 10 ml of air were extracted using a 10‐ml syringe. The indocyanine green solution was pushed to the front end of the syringe and injected (along with air) into the bronchus via the sheath tube. Next, 50 ml of air was rapidly injected via the sheath tube using a 50‐ml syringe, and indocyanine green solution was blown into the bronchial endings and alveoli. Similarly, other nodules in the ipsilateral or contralateral lung were localized successively (Figure 3).

### Single‐port video‐thoracoscopic wedge resection of lung

After methylene blue localization, patients were sent to the operating room for double‐lumen endotracheal intubation and general anesthesia. For patients receiving localization under VNB, the localization and surgery were performed in the same operating room. After localization, the blocking tube was introduced through endotracheal intubation. After ventilation with pure oxygen for 5 min, the main bronchus on the surgical side was blocked, followed by one‐lung ventilation and opening of the lumen of the blocking tube to exhaust the air in the lung on the surgical side. The surgery for pulmonary nodules on each side of the chest was performed via a 2–3 cm small incision at the fourth (upper lobe) or fifth (middle and lower lobes) intercostal space of the midaxillary line. During surgery, the relative position of the pulmonary nodules and the stained visceral pleura of the patients undergoing methylene blue localization were determined based on CT images after localization. Moreover, the visceral pleura corresponding to the pulmonary nodules was marked using an electric knife.

For localization under VNB, the stained visceral pleura observed via infrared fluorescence thoracoscopy (high‐definition fluorescence navigation endoscope system, Guangdong Opus Mandi Technology Co., Ltd) was chosen as the location of the pulmonary nodules. The nodules and their surrounding 1–2 cm lung tissue were wedge‐shaped resected using a linear cutting stapler. The localization failed if no stained visceral pleura was observed during the surgery. At this time, the pulmonary nodules were localized according to the preoperative 3D reconstructed image of the lung, and wedge resection of the lung was performed. Subsequently, the wedge‐shaped resected lung tissue specimens were cut open to search for the pulmonary nodules. If no nodule was found in the resected lung tissues, the location of the pulmonary nodules was further determined according to the preoperative 3D reconstructed image of the lung. In addition, the resection was extended or a segmental pneumonectomy was performed until pulmonary nodule resection. If the resected pulmonary nodules were close to the cutting edge, the cutting edge was considered insufficient, and the lung tissue resection was extended to a safe cutting edge. The resected specimens were sent for rapid frozen section pathological examination to clarify the pathological diagnosis and verify a negative cutting edge. When suturing the incision, intrathoracic gas was eliminated using a No. 18 sputum suctioning tube and the chest tube was not indwelt routinely.

### Observation indicators

1. Patient characteristics: Sex, age, number of pulmonary nodules resected simultaneously, and unilateral/bilateral lung surgery.
2. Pulmonary nodule features: Pulmonary nodule size, distance between pulmonary nodules and the visceral pleura, pulmonary nodule consolidation‐to‐tumor ratio (CTR), location of pulmonary nodules in the pulmonary lobe, and pulmonary nodule postoperative pathology.
3. Localization: (1) Localization time, methylene blue group: time from the first CT scanning to the completion of localization and the patient being removed from the scanning bed; virtual navigation group: time from the bronchoscope entering the endotracheal intubation to the completion of localization and bronchoscope withdrawal. (2) Pain score during localization: visual analog score (VAS) of pain experienced by patients who received preoperative CT‐guided percutaneous lung puncture localization. Patients who received VNB‐guided localization did not experience pain because the localization was performed after anesthesia. (3) Surgical duration: time from skin cutting to pulmonary nodule removal; waiting time for intraoperative rapid freezing pathology was not considered because not all patients needed to wait for intraoperative rapid freezing pathological results. (4) Complication incidence: complications caused by pulmonary nodule localization. (5) Visceral pleural staining rate after localization: the ratio of pleural staining observed during surgery indicated the localization success rate. (6) Pulmonary nodule primary resection success rate: defined as the number of pulmonary nodules resected at one time according to the localization, and intraoperative fast frozen pathology confirmed cutting edge was sufficient, without further extended resection or segmental pneumonectomy required. (7) Volume of resected lung tissue: the volume of lung tissue resected by wedge resection of each lung nodule, which was roughly calculated by multiplying three values of length, width and thickness of lung tissue specimens measured by the pathology department in this study. Although not precise enough, it is sufficient to compare the volume of resected lung tissue in the two localization methods.

### Statistical analysis

Normally distributed data were expressed as the mean ± standard deviation (x¯ ± *s*) and analyzed using the independent sample *t*‐test. Categorical data were expressed as *n* (%) and analyzed using the chi‐square test. Fisher's exact test was used if the expected frequency was <5. Multivariate analysis was performed using multivariate analysis of variance. An *α* value of 0.05 was considered statistically significant. Statistical analysis was performed using the R programming language (R Core Team, 2000).

## RESULTS

### Patient characteristics

The methylene blue group comprised 39 patients, with 119 pulmonary nodules resected. The virtual navigation group comprised 20 patients, with 67 pulmonary nodules resected. Sex, age, number of pulmonary nodules resected simultaneously, and unilateral/bilateral lung surgery did not differ significantly between the two groups (*p* > 0.05) (Table 1).

### Pulmonary nodule features

No significant differences were found in pulmonary nodule size, distance between pulmonary nodules and the visceral pleura, pulmonary nodule CTR, location of pulmonary nodules in the pulmonary lobe, or pulmonary nodule postoperative pathology between the two groups (*p* > 0.05) (Table 1).

### Localization and surgery

Localization time: Multivariate analysis of variance revealed that the localization time of the virtual navigation group was often significantly shorter than that of the methylene blue group, regardless of unilateral or bilateral multiple nodules (*p* < 0.05). In addition, the average localization time of patients with multiple nodules in both lungs differed remarkably between the virtual navigation and methylene blue groups, and the difference increased with an increasing number of nodules.

Surgical duration: Multivariate analysis of variance revealed no significant difference in surgical duration between the two groups, regardless of unilateral or bilateral lung surgery (*p* > 0.05). However, the surgical duration was found to be significantly increased with an increase in the number of simultaneously resected nodules (*p* < 0.05).

Incidence of complications: In the methylene blue group, 10/39 patients (25.64%) presented complications, all of which were pneumothorax. One patient with multiple nodules in both lungs suffered from bilateral pneumothorax. All patients with pneumothorax did not need closed drainage before surgery. No complications were found in the virtual navigation group.

Staining rate of the visceral pleura: Pleural staining was observed intraoperatively in all nodules after localization in the methylene blue group. No pleural staining was detected during the surgery in two nodules after localization in the virtual navigation group. The pulmonary nodules were successfully resected after localization by 3D lung surface projection. There was no significant difference in the staining rate of the visceral pleura after localization between the two groups (*p* > 0.05).

Success rate of primary pulmonary nodule resection: All pulmonary nodules were successfully resected in both groups. No significant difference was found in primary pulmonary nodule resection (*p* > 0.05). Two patients in the virtual navigation group and one in the methylene blue group received extended pulmonary resection owing to the long distance between the localization point and the actual position of the pulmonary nodule, short range of the primary pulmonary resection, and the cutting edge being close to the nodule.

**FIGURE 4:**
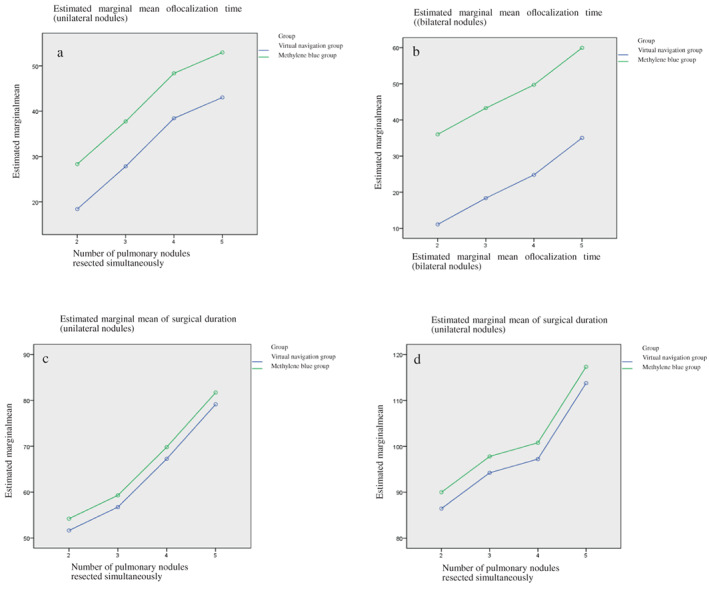
The relationship between localization time, surgical duration, and the number of pulmonary nodules resected simultaneously in the two groups. (a) Localization time of unilateral pulmonary nodules. (b) Localization time of bilateral pulmonary nodules. (c) Surgical duration of unilateral pulmonary nodules. (d) Surgical duration of bilateral pulmonary nodules

Volume of resected lung tissue: In the wedge resection of a pulmonary nodule, patients in the virtual navigation group had to resect larger lung tissue than patients in the methylene blue group (*p* < 0.05) (Table 2 and Figure 4).

**TABLE 2:** Statistical analysis results of observation indicators in the two groups

Observation indicators			Virtual navigation group (*x¯* ± *s*)	Methylene blue group (*x¯* ± *s*)	*t*/*χ* ^2^/*F*	*p*
Localization time (min)	Unilateral nodules	Total	26.71 ± 5.676 (*n* = 14)	37.22 ± 12.296 (*n* = 27)	Calibration model: *F* = 29.037	0.000
		Two nodules	21.83 ± 1.941 (*n* = 6)	26.45 ± 6.502 (*n* = 11)	*F*(group) = 23.724	0.000
		Three nodules	27.80 ± 1.643 (*n* = 5)	37.75 ± 6.964 (*n* = 8)	*F*(number of pulmonary nodules resected) = 29.097	0.000
		Four nodules	30.00 (*n* = 1)	49.57 ± 2.936 (*n* = 7)		
		Five nodules	37.00 ± 4.243 (*n* = 2)	65.00 (*n* = 1)		
	Bilateral nodular	Total	28.83 ± 4.956 (*n* = 6)	45.92 ± 10.689 (*n* = 12)	Calibration model: *F* = 14.739	0.000
		Two nodules	–	36.00 ± 1.414 (*n* = 2)	*F*(group) = 53.677	0.000
		Three nodules	23.00 (*n* = 1)	42.50 ± 6.892 (*n* = 6)	*F*(number of pulmonary nodules resected) = 8.660	0.002
		Four nodules	27.50 ± 3.536 (*n* = 2)	47.00 ± 1.414 (*n* = 2)		
		Five nodules	31.67 ± 4.933 (*n* = 3)	65.00 ± 4.243 (*n* = 2)		
Pain score during localization			–	2.67 ± 1.151	–	
Surgical duration (min)	Unilateral nodules	Total	58.50 ± 11.494 (*n* = 14)	60.78 ± 10.924 (*n* = 27)	Calibration model: *F* = 11.979	0.000
		Two nodules	52.50 ± 5.244 (*n* = 6)	53.73 ± 3.197 (*n* = 11)	*F*(group) = 0.964	0.333
		Three nodules	56.80 ± 10.872 (*n* = 5)	59.25 ± 6.882 (*n* = 8)	*F*(number of pulmonary nodules resected) = 15.697	0.000
		Four nodules	65.00 (*n* = 1)	70.14 ± 12.267 (*n* = 7)		
		Five nodules	77.50 ± 10.607 (*n* = 2)	85.00 (*n* = 1)		
	Bilateral nodular	Total	105.00 ± 9.920 (*n* = 6)	100.25 ± 9.469 (*n* = 12)	Calibration model: *F* = 25.154	0.000
		Two nodules	–	90.00 ± 0.000 (*n* = 2)	*F*(group) = 2.808	0.118
		Three nodules	95.00 (*n* = 1)	97.67 ± 3.327 (*n* = 6)	*F*(number of pulmonary nodules resected) = 31.360	0.000
		Four nodules	97.50 ± 2.121 (*n* = 2)	100.50 ± 0.707 (*n* = 2)		
		Five nodules	113.33 ± 5.774 (*n* = 3)	118.00 ± 7.071 (*n* = 2)		
Complication incidence % (*n*/*n*)			0% (0/20)	25.64% (10/39)	6.175	0.012
Staining rate of the visceral pleura % (*n*/*n*)			97.01% (65/67)	100% (119/119)	3.591	0.129
Success rate of primary pulmonary nodule resection % (*n*/*n*)			97.01% (65/67)	99.16% (118/119)	1.243	0.295
Postoperative pathology % (*n*)		AAH	5.97% (4/67)	3.36% (4/119)	0.947	0.623
		AIS	47.76% (32/67)	45.38% (54/119)		
		MIA	46.27% (31/67)	52.10% (62/119)		
Volume of resected lung tissue (cm^3^)			6.82 ± 1.607	4.52 ± 1.409	10.129	0.000

*Abbreviations*: AAH, atypical adenomatous hyperplasia; AIS, adenocarcinoma in situ; MIA, minimally invasive adenocarcinoma.

## DISCUSSION

ENB and VNB are increasingly used in clinical practice. The application of ENB for the localization of pulmonary nodules has also been reported frequently.^21^, ^22^, ^23^, ^24^, ^25^, ^26^, ^27^, ^28^ Although doctors in different hospitals have differences in using VNB to locate pulmonary nodular nodes, for example Hirokazu Yamaguchi^29^ used multi‐point positioning combined with 3D reconstruction and Samy Lachkar^30^ used VBN combined with ultrasound probe to locate pulmonary nodular nodes, but all showed good results. Jianxing He's team in the Department of Thoracic Surgery of the First Affiliated Hospital of Guangzhou Medical University reported 173 lung nodule localization cases under ENB, the study with the largest sample size so far.^31^ This study suggests that pulmonary nodule localization under ENB is an accurate and effective method with a short surgical duration and few complications. Both ENB and VNB can be used to localize pulmonary nodules and are used in our department.

In some cases, the localization effects of the two methods are similar. However, the cost of the localizing probe required for ENB is higher than that of the sheath tube for the injection needed for VNB, therefore more doctors choose VNB. The present study also used VNB for observation.

The commonly used agents for pulmonary nodule localization under VNB include methylene blue and indocyanine green.^30^, ^32^ In clinical application, only the sheath tube can enter bronchi below grade 6 for methylene blue injection for the lung surface to be well stained. However, in some cases, this is not possible under bronchoscopy. Because of its certain penetrability, indocyanine green injected into the peripheral bronchi can develop well without diffusion into the visceral pleura,^33^ therefore we primarily used indocyanine green for localization.

This retrospective study showed that indocyanine green injection under VNB could achieve the same effect in pulmonary nodule localization as classical methylene blue injection under CT‐guided percutaneous lung puncture. No significant differences were observed in the success rate of pulmonary nodule primary resection or surgical duration between the two groups. However, in patients with multiple nodules undergoing simultaneous surgery, the localization time was significantly shorter and complication incidence was significantly lower in the virtual navigation group than in the methylene blue group. This difference is mainly caused by the localization of multiple nodules, especially nodules in both lungs, requiring multiple CT scans and positional changes, thereby considerably increasing time consumption in methylene blue injection under percutaneous puncture. Moreover, pneumothorax is an inevitable risk because of the damage to the visceral pleura caused by the puncture needle. Bilateral pneumothorax after localization may lead to severe accidents, especially in patients with bilateral pulmonary nodules undergoing simultaneous surgery. Furthermore, the localization by indocyanine green injection under VNB is completed under anesthesia to mitigate the tension, anxiety, and pain of patients in preoperative localization.

There are some limitations in the localization by indocyanine green injection under VNB. In this study, two pulmonary nodules in the virtual navigation group showed no fluorescence after localization under fluorescent thoracoscopy. The reason is that the target bronchi of these nodules are thin and tortuous. The bronchoscope can enter only a grade 3 bronchus, while the sheath tube can enter only a grade 4 bronchus. After indocyanine green injection, the drug flows back into the superior bronchus rather than entering the peripheral bronchus or alveoli. Therefore, to prevent dissatisfaction in localization under VNB, a spare localization method is needed during the surgery. Our department routinely used patients' lung CT scans for 3D reconstruction before surgery to localize the pulmonary nodules by 3D lung surface projection in the case of localization failure under VNB. In addition, the farther the pulmonary nodules are from the target bronchus in preoperative CT, the farther the pulmonary nodules are from the fluorescent staining center in surgically resected specimens, and thus the lower the accuracy of localization.

Furthermore, when localizing pulmonary nodules under VNB, indocyanine green is injected into the distal end of grade 5 and 6 bronchi. A piece of lung tissue belonging to the bronchus is developed by fluorescence. Consequently, only pulmonary nodules can be localized in this piece of lung tissue, and only the completely resected lung tissue piece can ensure that the pulmonary nodules are resected. Methylene blue localization is a fixed‐point method that localizes pulmonary nodules at a certain point. With accurate localization, the pulmonary nodules can be removed by resecting the localization point and surrounding small pieces of lung tissue, therefore the resected lung tissue localization with indocyanine green was larger than that with methylene blue. We believe that by using 3D lung reconstruction and tracheal drainage, we can reconstruct the lung unit to which the target bronchus belongs as well as the location of this lung unit where pulmonary nodules are located. The location of pulmonary nodules can be determined based on the 3D reconstructed images after visualizing the stained lung units via fluorescence imaging during surgery to achieve a more accurate and smaller resection.

## CONCLUSIONS

For patients with multiple pulmonary nodules undergoing simultaneous surgery, indocyanine green injection under VNB can achieve a similar effect in pulmonary nodule localization as classical methylene blue injection under CT‐guided percutaneous lung puncture, with a shorter localization time and fewer complications. In addition, patients need not experience pain and fear during localization. Combined with 3D lung reconstruction, indocyanine green injection under VNB may contribute to more accurate localization.

## CONFLICTS OF INTEREST

All authors have completed the ICMJE uniform disclosure form. The authors have no conflicts of interest to declare.

## REPORTING CHECKLIST

The authors have completed the STROBE reporting checklist.

## DATA SHARING STATEMENT

N/A

## PEER REVIEW FILE

Available at https://doi.org/10.21037/tlcr-21-699.

## ETHICAL STATEMENT

The authors are accountable for all aspects of the work in ensuring that questions related to the accuracy or integrity of any part of the work are appropriately investigated and resolved. The study was conducted in accordance with the Declaration of Helsinki (as revised in 2013). The study was approved by the Ethics Committee of the Xiamen Humanity Hospital of Fujian Medical University (No. 2020K‐43) and individual consent for this retrospective analysis was waived.

## OPEN ACCESS STATEMENT

This is an Open Access article distributed in accordance with the Creative Commons Attribution‐NonCommercial‐NoDerivatives 4.0 International License (CC BY‐NC‐ND 4.0), which permits the noncommercial replication and distribution of the article with the strict provision that no changes or edits are made and the original work is properly cited (including links to both the formal publication through the relevant DOI and the license).

## CONTRIBUTIONS

(i) Conception and design: Qingjie Yang; (ii) Administrative support: Mingqiang Kang and Kaibao Han; (iii) Provision of study materials or patients: Shenghua Lv and Qingtian LI; (iv) Collection and assembly of data: Shenghua Lv and Qingtian Li; (v) Data analysis and interpretation: Qingjie Yang, Xiaoyan Sun, and Xinhai Feng; (vi) Manuscript writing: All authors; (vii) Final approval of manuscript: All authors.
